# Vitamin D deficiency and fatigue: an unusual presentation

**DOI:** 10.1186/s40064-015-1376-x

**Published:** 2015-10-07

**Authors:** Kevin Johnson, Maryam Sattari

**Affiliations:** Department of Medicine, University of Florida College of Medicine, 1600 SW Archer Rd, PO Box 100277, Gainesville, FL 32610 USA

**Keywords:** Vitamin D deficiency, Fatigue, Excessive daytime sleepiness

## Abstract

Fatigue is a vague but common complaint that is poorly characterized by physicians as well as patients. While fatigue may result from a number of different etiologies, at the present time, a comprehensive approach to each patient with fatigue does not include routine measurement of serum vitamin D levels. A 61-year-old man was evaluated for excessive daytime fatigue. No features characteristic for depression, sleep apnea, or narcolepsy were present. A comprehensive work-up, including thyroid function tests and testosterone levels, did not reveal any abnormalities. However, serum 25-hydroxyvitamin D level was low, at 18.4 ng/mL. Vitamin D supplementation was initiated. At follow-up in 3 and 12 months, the patient reported complete resolution of daytime fatigue, corresponding to an increase in his vitamin D levels. Possible mechanisms for clinical improvement include effects of vitamin D on components of inflammatory cascades, including tumor necrosis factor-alpha and prostaglandin D2, which result in decrease in central nervous system homeostatic sleep pressure. While more research is needed to determine if patients presenting with fatigue should be routinely screened for vitamin D deficiency, clinicians should consider obtaining vitamin D levels in patients with unexplained fatigue, nonspecific musculoskeletal pain, and risk factors for vitamin D deficiency.

## Background

Fatigue is a vague but common complaint that is poorly characterized by physicians as well as patients. While fatigue may result from a number of different etiologies, at the present time, a comprehensive approach to each patient with fatigue does not include routine measurement of serum vitamin D levels. Vitamin D refers to a group of fat-soluble secosteroid hormones, and is typically ingested in dietary sources or manufactured in the skin after exposure to sunlight (Holick 2007). Increasing evidence suggests that vitamin D has many roles beyond its classically described effects on calcium homeostasis and bone health (Holick 2007). Research suggests possible associations between suboptimal levels of vitamin D and development of various diseases, including pulmonary disorders (Black and Scragg 2005; Sita-Lumsden et al. 2007; Camargo et al. 2007; Devereux et al. 2007; Litonjua and Weiss 2007) chronic rhinitis (Abuzeid et al. 2012), tonsillar hypertrophy, (Nunn et al. 1986; Reid et al. 2011), metabolic syndrome (Botella-Carretero et al. 2007), type 2 diabetes (Mattila et al. 2007), hypertension (Forman et al. 2007), cancers of the breast, colon, and prostate (Garland et al. 2006), poor stress resilience (Bracha et al. 2004), depression (Berk et al. 2007), and cognitive decline (Przybelski and Binkley 2007). Vitamin D appears to be necessary for skeletal muscle as well and its deficiency has been associated with nonspecific musculoskeletal pain (Plotnikoff and Quigley 2003), chronic pain (Turner et al. 2008), low back pain (Lotfi et al. 2007), and myopathy (Boltan et al. 2007; Goldstein 2007; Prabhala et al. 2000). Some researchers have even suggested a link between vitamin D deficiency and all-cause mortality (Giovannucci 2007).

Vitamin D also has immunomodulatory activities (Holick 2007). Deficiency of vitamin D might be associated with diseases of immune dysregulation, one manifestation of which could be excessive daytime sleepiness (Zitterman and Gummert 2010; Hoeck and Pall 2011). We present a case of daytime fatigue in an otherwise healthy male who was found to be vitamin D deficient.

## Case presentation

A 61-year-old Caucasian man presented to primary care office with complaint of fatigue and daytime sleepiness, especially in the afternoons. His symptoms began gradually 2–3 months prior to presentation, insidiously worsening to the point that he began having functional difficulties with his normal tasks at work in the afternoons. He reported napping almost daily after work and even skipping some of his regular exercise sessions due to fatigue. He denied changes in his weight, new familial or occupational stressors, difficulty falling asleep, snoring, apnea, sleep disruptions, nocturnal awakenings, depression, or anxiety. In fact, his review of symptoms was only positive for chest pain that was worse in the afternoon when he felt tired. He reported good sleep hygiene and was able to get his customary 7–8 h of sleep each night. Before the onset of his symptoms, he had worked fulltime and exercised on an almost daily basis, without experiencing any difficulties. His past medical history was only significant for colon cancer, in remission since surgical resection and completion of systemic adjuvant chemotherapy in 2005 (7 years prior to presentation). He did not take any prescription medications and denied use of tobacco products, alcohol, or recreational drugs.

Physical exam revealed a pleasant male in no distress. Vital signs were within normal limits. His body mass index was 28. No significant abnormalities were detected on complete physical exam. Laboratory data, including thyroid stimulating hormone, liver function tests, and renal indices, were normal (Table 1). EKG and stress echocardiogram were normal. In absence of a common etiology explaining patient’s symptoms, serum 25-hydroxy vitamin D level was obtained and found to be low at 18.4 (normal range 30–80 ng/mL). Vitamin D replacement was initiated with ergocholecalciferol 50,000 international units (IU) weekly for 8 weeks, followed by vitamin D 1000 IU daily.

**Table 1:** Patient’s lab results on presentation

Lab	Value	Reference range
White blood cell	5.2	4.0–10.0 thousands/cu mm
Hemoglobin	13.9	13.0–16.0 g/dL
Sodium	142	136–145 mmol/L
Potassium	4.0	3.3–5.1 mmol/L
CO_2_	28	22–30 mmol/L
BUN	17	6–20 mg/dL
Creatinine	0.99	0.80–1.20 mg/L
Glucose	92	65–99 mg/dL
AST	14	0–37 U/L
ALT	15	0–41 U/L
Total CPK	128	30–170 U/L
Erythrocyte sedimentation rate	10	0–10 mm/h
Vitamin B12	1031	243–846 pg/mL
TSH	2.96	0.27–4.20 mL U/L
Testosterone free	47	47–244 pg/mL
CEA	2.1	0.0–4.3 ng/mL
Serum 25-hydroxy vitamin D	18.3	>29 ng/mL

Patient reported improvement of his fatigue and daytime sleepiness within 2 weeks of initiation of vitamin D supplementation and complete resolution of his symptoms within 3 months of vitamin D initiation. In follow-up visit in 3 months, he reported being able to perform his previous daily routine without difficulty. In addition to working full-time, he had resumed his exercise routine (3 sessions of resistance training and at least 6 sessions of 30–60 min of cardiovascular training a week) without experiencing any limitations. He denied chest pain or daytime napping. He stated that—aside from initiating vitamin D supplementation—no other circumstances in his life had changed since his initial evaluation: there had been no interval changes in other medications, diet, social activities, caffeine use, stress level, or work. He continues to feel well and remains completely symptom-free to date. His repeat 25-hydroxy vitamin D levels were 27.2 ng/mL after 3 months of vitamin D supplementation and 32.2 ng/mL after 12 months (Table 2).

**Table 2:** Patient’s serum 25-hydroxy vitamin D levels

Time	Vitamin D level (ng/mL)
Baseline	18.4
3 months	27.2
12 months	32.2

## Discussion

The exact mechanism for the improvement in this patient’s fatigue after identification and treatment of vitamin D deficiency is not known. To our knowledge, this is the second reported case of daytime fatigue and sleepiness resolving upon remediation of vitamin D deficiency. McCarty has previously reported a case of excessive daytime sleepiness and musculoskeletal pain in a 28-year-old African American female that improved with replacement of vitamin D (McCarty 2010). This patient underwent an overnight polysomnogram (PSG) before and after vitamin D repletion (McCarty 2010). While a full sleep evaluation before vitamin D repletion revealed the presence of heavy daytime napping and pervasive fatigue, the PSG did not show evidence of sleep disordered breathing or a sleep-related movement disorder (McCarty 2010). Post-replacement PSG did not show a decrease in episodes and duration of wake after sleep onset or an improvement in sleep continuity, but did reveal an interval decrease in stage N3 sleep, suggesting a reduction in homeostatic sleep pressure following vitamin D replacement (McCarty 2010).

**Fig. 1:**
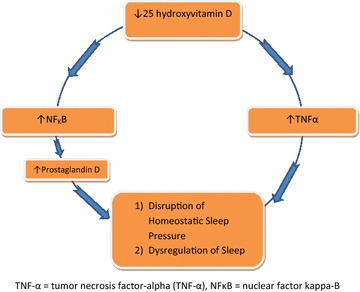
Proposed relationship between vitamin D and sleep regulation

McCarty postulated that vitamin D deficiency may contribute to symptoms of sleepiness via components of inflammatory cascades, including known sleep regulating substances (McCarty et al. 2012). For example, there is an inverse relationship between levels of tumor necrosis factor-alpha (TNF-α) and serum 25-hydroxyvitamin D (Fig. 1) (Peterson and Heffernan 2008). TNF-α has been implicated in the sleepiness associated with obstructive sleep apnea (Peterson and Heffernan 2008; Churchill et al. 2008). Vitamin D deficiency has also been associated with upregulation of nuclear factor kappa-B (NFĸB) (Jablonski et al. 2011), which is responsible for the regulation of numerous substances known to exert homeostatic sleep pressure, including prostaglandin D2 (Chen et al. 1999; Krueger et al. 2009). Prostaglandin D2 functions as a physiologic regulator of sleep and affects the central nervous system homeostatic sleep pressure (Fig. 1) (McCarty et al. 2012).

Interestingly, the only identifiable potential risk factor our patient had for vitamin D deficiency was skin-protective behavior that consisted of sun avoidance and use of SPF protection. Though vitamin D deficiency is commonly understood to be disproportionately represented in underserved populations (Kakarala et al. 2007), patients residing in northern latitudes (Webb et al. 1988), individuals with darker skin tones (Matsuoka et al. 1991, 1995), the elderly (Holick et al. 2005), the obese (Wortsman et al. 2000), and pregnant or lactating women (Lee et al. 2007), its prevalence among the general population is also increasing (Faiz et al. 2007; Zargar et al. 2007). In fact, vitamin D deficiency or insufficiency is estimated to affect over a billion persons worldwide (Holick 2007). Increased awareness of potential dangers of sun exposure, skin-protective behavior, and urbanization of the population are thought to be some of the factors underlying the increase in prevalence of vitamin D deficiency and insufficiency (Holick 2007). It is of note that this patient’s serum parathyroid hormone level was not checked and therefore, normocalcemic primary hyperparathyroidism cannot be ruled out as a contributing factor to his symptoms.

## Conclusions

Our case lends support to the one presented by McCarty that vitamin D deficiency might be an unrecognized and easily reversible etiology of fatigue. Although a causal relationship cannot be confirmed by this case alone, the temporal relationship as well as biological plausibility makes this a possibility. While further study is needed to elucidate the possible mechanism for this association, and whether widespread screening for vitamin D deficiency among patients complaining of daytime sleepiness/fatigue is warranted, clinicians should consider obtaining serum vitamin D levels in patients who present with daytime sleepiness/fatigue, nonspecific musculoskeletal pain, and risk factors for vitamin D deficiency.
